# Endothelial Dysfunction and Inflammation Precedes Elevations in Blood Pressure Induced by a High-Fat Diet

**DOI:** 10.5935/abc.20180086

**Published:** 2018-07

**Authors:** Jorge Camargo Oishi, Cynthia Aparecida Castro, Karina Ana Silva, Victor Fabricio, Evelin Capelari Cárnio, Shane A. Phillips, Ana Claudia Garcia de Oliveira Duarte, Gerson Jhonatan Rodrigues

**Affiliations:** 1 Universidade Federal de São Carlos (UFSCar), São Paulo, SP - Brazil; 2 Universidade de São Paulo (USP), Ribeirão Preto, SP - Brazil; 3 University of Chicago Medical Center, Chicago, Illinois - USA

**Keywords:** Hypertension, Endothelium / abnormalities, Diet, High-Faties, Nitric Oxide, Dyslipidemias

## Abstract

**Background:**

Obesity leads to a chronic inflammatory state, endothelial dysfunction and hypertension.

**Objective:**

To establish the time-course of events regarding inflammatory markers, endothelial dysfunction, systolic blood pressure (SBP) in obesity in only one experimental model.

**Methods:**

We fed male Wistar rats (eight-week age) with a standard diet (Control - CT, n = 35), or palatable high-fat diet (HFD, n = 35) for 24 weeks. Every six weeks, 7 animals from each group were randomly selected for euthanasia. SBP and serum levels of interleukin-6, tumor necrosis factor-α, C-reactive protein, adiponectin and nitric oxide were determined. Endothelial and vascular smooth muscle functions were determined in dissected aorta and lipid peroxidation was measured. Statistical significance was set at p < 0.05.

**Results:**

Levels of pro-inflammatory cytokines began to increase after six weeks of a high-fat diet, while those of the anti-inflammatory cytokine adiponectin decreased. Interestingly, the endothelial function and serum nitric oxide began to decrease after six weeks in HFD group. The SBP and lipid peroxidation began to increase at 12 weeks in HFD group. In addition, we showed that total visceral fat mass was negatively correlated with endothelial function and positively correlated with SBP.

**Conclusion:**

Our results show the time-course of deleterious effects and their correlation with obesity.

## Introduction

Currently, obesity and associated comorbidities are one of the major health problems in developed and developing countries, reducing both the quality and quantity of life and increasing the risk of mortality.^1^ Obesity is characterized by excessive fat tissue storage and is strongly associated with the development of cardiovascular diseases, dyslipidemia and hypertension. There is an associated pro-inflammatory environment that appears to worsen cardiovascular outcomes^2,3^ and according to the World Health Organization,^4^ cardiovascular diseases are currently one of the major causes of mortality in the world.

A great number of metabolic disorders are caused by obesity; among them endothelial dysfunction plays an important role in the development of insulin resistance and hypertension.^5^ Almost thirty-five years ago, it was discovered that endothelial cells could modulate relaxations and contractions of the underlying vascular smooth muscle, which allowed for the concept that vascular tonus control is endothelium-dependent of the underlying vascular smooth muscle.^6-8^

The endothelium produces several "relaxing factors" (EDRFs, endothelium-derived relaxing factors), hyperpolarizing factors (EDHFs), as well as contractile factors (EDCFs). Through a fine balance between the release of EDRFs and EDCFs, the endothelium plays a vital role in maintaining circulatory homeostasis. Any change in this balance may result in endothelial dysfunction.^5,8^

Previous studies have demonstrated the onset of hypertension and endothelial dysfunction in obesity induced by a high-fat diet.^9,10^ However, whether and in which order they appear has not been well defined and the temporal relationships between weight gain, endothelial dysfunction and blood pressure following a high-fat diet have not been determined. Therefore, the aim of the present study was to determine the time course of inflammation, endothelial dysfunction and the increase in blood pressure following a high-fat diet designed to induce obesity.

## Methods

### Animals and dietary treatments

The experimental protocol was in accordance with the guidelines of the Brazilian College for Animal Experimentation (COBEA) and was approved by the Ethical Committee of the Federal University of São Carlos (026/2013).

Seventy male (8-week-old) Wistar rats (250-300 g) were assigned to two experimental groups with food and water *ad libitum* for 24 weeks: Control (CT, n = 35) was fed a standard diet or HFD (n = 35) fed with high-fat diet, that consisted of a standard rat diet plus peanuts, milk chocolate, and biscuits at a proportion of 3:2:2:1 as previously described.^11^ Standard diet and high-fat diet contained, respectively, 20/20% of protein, 4.5/20% of fat and 55/40% of carbohydrate.^11^ The caloric values of the diets were approximately 4.07 kcal/g for the standard diet and 5.12 Kcal/g for HFD. At time 0 and after every 6 weeks, 7 rats from CT and 7 from HFD group were randomly euthanized, and blood was collected for experimental analysis.

### Blood pressure measurements in Conscious Rats

Indirect systolic blood pressure (SBP) was measured two days before euthanasia every 6 weeks using tail-cuff plethysmography (Power Lab 8/35, AD Instruments, Pty Ltda, CO), as described by Rodrigues et al.^12^ Mean SBP was calculated from an average of four successive measurements in each animal.

### Vascular reactivity studies

The animals were anaesthetized with isoflurane and euthanized by decapitation. Thoracic aortas were isolated and cleaned of adherent connective tissues, and placed in a Krebs solution, as described previously.^13^ Aortas were carefully mounted as ring preparations (≅ 4 mm in length) and placed in bath chambers containing Krebs solution at 37ºC continuously bubbled with 95% O_2_ and 5% CO_2_, pH 7.4 in an isometric myograph (model 610 DMT-USA) and recorded by a PowerLab8/SP data acquisition system (AD Instruments Pty Ltd., Colorado). The aortic rings were submitted to a tension of 1.5 g, which was readjusted every 15 min during a 60 min equilibration period before adding the given drug. Experiments were done in aortic rings with intact endothelium and also in denude endothelium aortic rings. Endothelial integrity was assessed by the degree of relaxation induced by 1 µmol/l acetylcholine (ACH) in the presence of contractile tone induced by phenylephrine (0.1 µm/l). The ring was considered with intact endothelium if the relaxation with acetylcholine was higher than 80%. In endothelium-denuded aortas, the relaxation to ACH was less than 5%. After the endothelial integrity test, aortic rings were pre-contracted with phenylephrine (100 nM). When the plateau was reached, concentration-effect curves to acetylcholine (0.1nM to 0.1mM) in intact endothelium aortic rings or concentration-effect curves for NO donor sodium nitroprusside (SNP) in denude endothelium aortic rings were constructed. Concentration curves were fitted with a sigmoidal dose-response equation which disclosed the maximal effect (MaxE) and the negative logarithm of the agonist that produces half-maximal response (pD2) using GraphPad Prism (GraphPad Software In, USA).

### Body fat composition

Visceral adipose tissue (VAT) was dissected (mesenteric, epididymal and retroperitoneal white adipose tissues) and weighed to evaluate central adiposity.

### Aorta lipid peroxidation (Ferrous oxidation-Xylenol Orange - FOX)

Thoracic aortas were isolated and cleaned of adherent connective tissues. The methodology was described by Jiang et al.^14^ The ferrous oxidation−xylenol orange (FOX), measures lipid peroxides (cumene hydroperoxide - CHP), one of the main products of lipid peroxidation. For the standard assay, the following reagents were added sequentially: 0.25 mM FeSO_4_, 25 mM H2SO_4_, 0.1 mM xylenol orange, and water to a total of 0.9 ml. A sample of tissue extract (20-100 µL) was added, and the final volume was adjusted to 1 ml with water. Blanks were prepared by replacing tissue extract with water. Samples were incubated at room temperature until the reaction was complete (40 min), and absorbance at 560 nm was measured.

### Serum nitrite and nitrate (NOx)

Serum nitric oxide levels were obtained by measuring the serum concentrations of its stable end-products nitrite (NO_2_^-^) and nitrate (NO_3_^-^), collectively known as NOx, as described previously.^15^ The NO/ozone chemiluminescence method was performed using the NO Analyzer 280i (Sievers, Boulder, CO, USA).

### Determination of adiponectin and inflammatory cytokines

Quantification of adiponectin and inflammatory cytokines tumor necrosis factor-α (TNF-α), interleukin 6 (IL-6) and C-reactive protein (CRP) in serum was performed using the enzyme-linked immunosorbent assay (ELISA) kit. IL-6 and TNF-α were evaluated using commercial OptEIA kits (BD Biosciences, Pharmingen, USA). Adiponectin and CRP were analyzed using Duo Set kits (R&D Systems, USA). All kits were used according to the manufacturers' instructions, and the results were expressed in pg/mL for all cytokines evaluated.

### Morphological and histological evaluation

Aorta segments were quickly cleaned from the surrounding tissues and blood, cut into rings fixed in formalin 37% and embedded in paraffin blocks. Later, 4-µm thick sections were cut with a microtome (Leitz 1512, IMEB, USA), placed onto glass microscope slides and stained with hematoxylin and eosin using the standard methods. Images of transverse sections of the arterial segments were captured using a camera connected to an optical microscope (Leica DM 2000). External diameter (ED) was obtained by measuring the surfaces of the adventitia and internal diameter (ID) from the endothelium surface. The media thickness was obtained by dividing the difference ED - ID by 2 (δ = ED - ID/2). The media/lumen ratio was calculated from the area data. The images were analyzed using the ImageJ analysis software, as described previously.^16^

### Statistical analysis

The normality of distribution (of all quantitative and continuous variables) was checked using the Kolmogorov-Smirnov test. A sample of 7 animals in each group was required to provide 85% statistical power with a two-tailed alpha of 0.05 for pD2 and 90% for all other variables analyzed in this study. Differences between the CT and HFD groups were compared using two-way repeated measures analysis of variance ANOVA. When differences were indicated, a Newman-Keuls post hoc analysis was used with a statistical significance set at p < 0.05. These data were expressed as mean ± SD (Statistica software 7.0, StatSoft. Inc, USA). Vascular reactivity data of pD2 and Emax were expressed as mean ± SD with a statistical significance set at p < 0.05 (Graphpad Prism 3.0). Pearson correlation was made between pD2 and the SBP, pD2 and VAT, blood pressure and VAT, IL-6 and pD2, TNF-α and pD2, CRP and pD2 and between adiponectin and pD2, with a statistical significance of 5%.

## Results

### Total visceral adipose tissue

**Figure 1:**
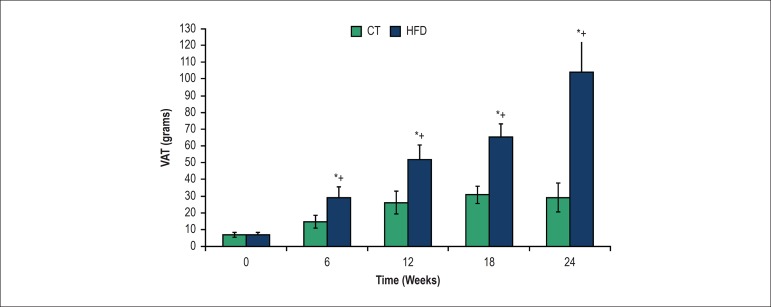
Visceral adipose fat (VAT) in control (CT) and high-fat diet (HFD) groups over the weeks. *P < 0.05, compared with CT; + p < 0.05, within-group comparison (0 vs. 6, 6 vs. 12, 12 vs. 18, 18 vs. 24 weeks). Seven rats from each group were compared at each time point.

The sum of the weight of the retroperitoneal, visceral and epididymal adipose tissues - (VAT) was higher in HFD than in the CT group at 6 weeks. At 24 weeks, fat weight was 300% higher in the HFD than the CT group. VAT in the CT group increased at 12 weeks compared to 6 weeks, but remained unchanged for the rest of the experimental period (Figure 1).

### Inflammatory status

**Figure 2:**
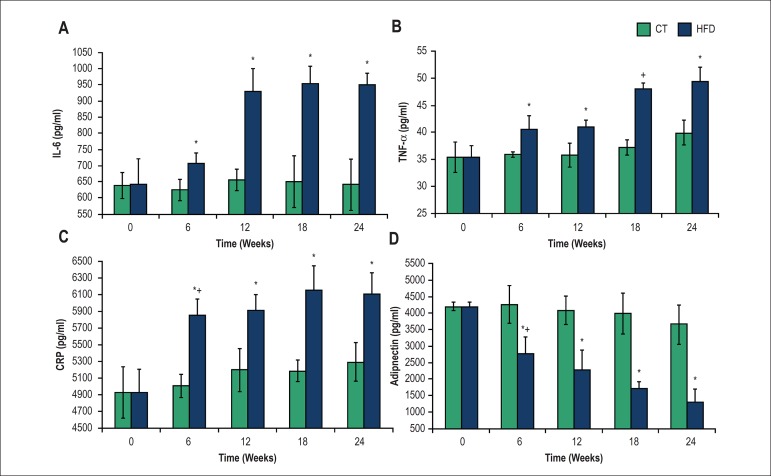
Serum interleukin-6 (IL-6) (A), tumor necrosis factor-α (TNF-α) (B), C-reactive protein (CRP) (C) and adiponectin (D) in the control (CT) and high-fat diet (HFD) groups over time. *P < 0.05, CT compared with HFD group; + p < 0.05, within-group comparison (0 vs. 6, 6 vs. 12, 12 vs. 18, 18 vs. 24 weeks). Seven rats from each group were compared at each time point

The inflammatory cytokines IL-6, TNF-α and CRP were increased in serum of HFD animals in 6, 12, 18 and 24 weeks when compared to the CT group (Figure 2 A, B, C). On the other hand, the levels of serum adiponectin decreased in the HFD group after 6, 12, 18 and 24 weeks of the experimental protocol (Figure 2 D). In the CT group, no changes were found in these cytokine levels.

### Vascular reactivity

**Figure 3:**
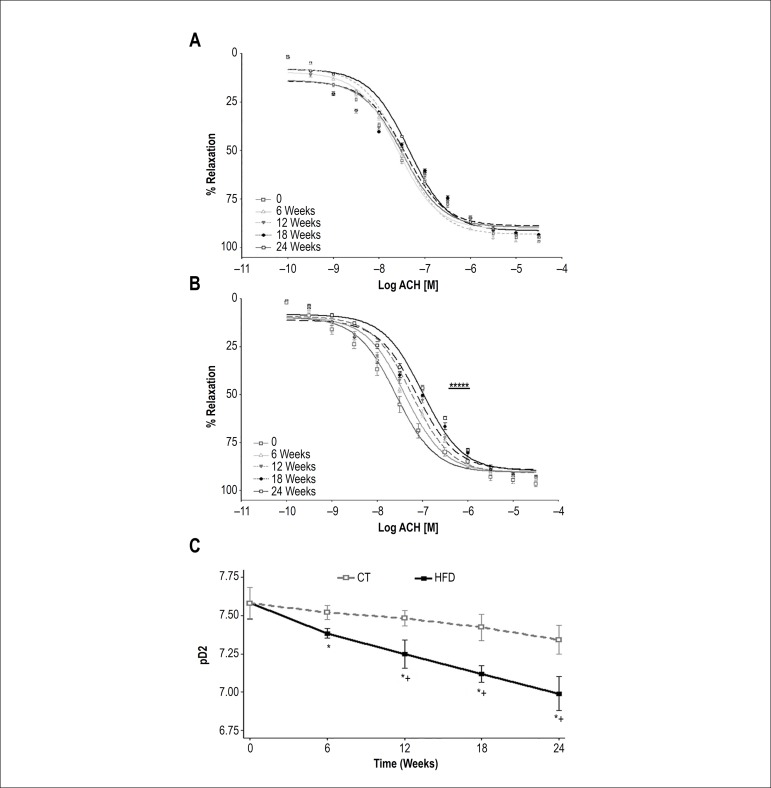
Concentration-response curve to acetylcholine (endothelium-dependent relaxation) in aortic rings of rats of the control (CT) (A) and high-fat diet (HFD) group (B) groups and half-maximal response pD2 (C) in both groups. *P < 0.05, CT compared with HFD group in each 6 weeks; + p < 0.05, within-group comparison (0 vs. 6, 6 vs. 12, 12 vs. 18, 18 vs. 24 weeks); seven rats from each group were compared at each time point

No differences were found (Figure 3A) in the endothelium-dependent relaxation induced by acetylcholine (pD2) in the CT group over the entire experimental period. On the other hand, the pD2 was impaired in aortas of obese animals at 6, 12, 18 and 24 weeks compared to CT rats. Moreover, we observed a decrease in pD2 throughout the experimental period in HFD group (Figures 3B, C).

No differences were observed in the maximum relaxant effect (MaxE) in both CT and HFD groups. In endothelium-denuded aortic rings, there were no differences in the pD2 and MaxE to endothelium-independent relaxation induced by SNP in the CT and HFD groups in all the weeks evaluated (Table 1).

**Table 1:** Half-maximal response (pD2) and maximal effect (MaxE) in aortic rings of the rats of the control (CT) and high-fat (HFD) groups. *P < 0.05, compared with CT group; + p < 0.05, within-group comparison (0 *vs*. 6, 6 *vs*. 12, 12 *vs*. 18, 18 *vs*. 24 weeks)

Weeks	Intact Endothelium				Denude Endothelium			
	pD2		MaxE(%)		pD2		MaxE(%)	
	CT	HFD	CT	HFD	CT	HFD	CT	HFD
0	7.58 ± 0.25	7.58 ± 0.22	90.67 ±7.40	90.87 ± 7.14	8.69 ± 0.13	8.68 ± 0.23	103.8 ± 2.77	104.6 ± 3.38
6	7.52 ± 0.07	7.37 ± 0.18*^+^	93.42 ±6.80	90.31 ± 7.64	8.67 ± 0.21	8.66 ± 0.22	98.3 ± 4.10	100.2 ± 7.67
12	7.48 ± 0.18	7.23 ± 0.11*^+^	89.17 ±8.80	90.90 ± 7.67	8.69 ± 0.10	8.71 ± 0.13	102.5 ± 2.48	103.9 ± 3.43
18	7.42 ± 0.22	7.12 ± 0.15*^+^	88.98 ±9.90	89.34 ± 10.05	8.71 ± 0.10	8.69 ± 0.07	105.8 ± 3.70	104.3 ± 1.85
24	7.34 ± 0.19	6.99 ± 0.23*^+^	91.46 ±6.61	89.80 ± 8.59	8.69 ± 0.07	8.68 ± 0.14	105.9 ± 2.98	105.9 ± 2.11

There was a strong negative correlation between pD2 and SBP (r = -0.722, p < 0.01). Moreover, we found a negative correlation between pD2 and VAT (r = -0.729, p < 0.01), between pD2 and inflammatory cytokines (pD2 and IL-6, r = -0.74; pD2 and TNF-α, r = -0.86; pD2 and CRP, r = -069, p < 0.05) and a positive correlation between pD2 and adiponectin (r = 0.77, p < 0.01).

### Serum nitric oxide (NO) and aorta lipid peroxidation

**Figure 4:**
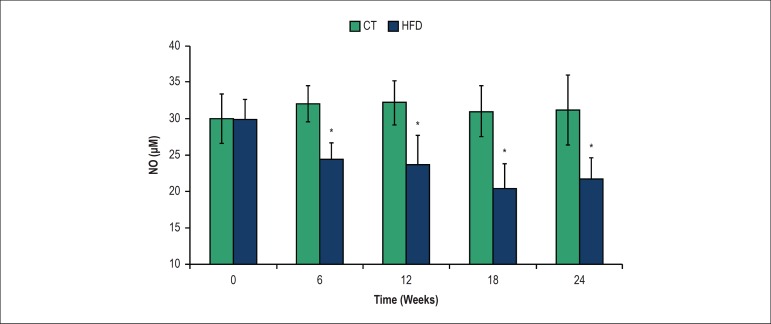
Serum nitric oxide (NO) concentration in rats of the control (CT) and high-fat diet (HFD) groups. *P < 0.05, compared with CT group; + p < 0.05, within-group comparison (0 vs. 6, 6 vs. 12, 12 vs. 18, 18 vs. 24 weeks); seven rats from each group were compared at each time point

By quantification of serum NO metabolites, we observed that NO level decreased at 6 weeks in HFD rats and remained lower throughout the experimental period when compared to the CT group. The time of experiment had no effect on NO concentrations in CT and the HFD groups (Figure 4).

**Figure 5:**
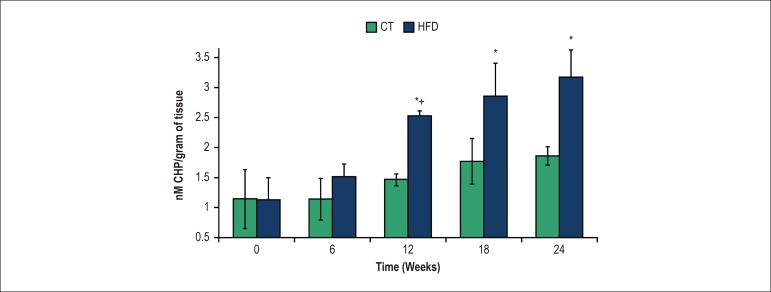
Lipid peroxidation in aortic rings from rats of control (CT) and high-fat diet (HFD) groups. *P < 0.05, compared with CT group; + p < 0.05, within-group comparison (0 vs. 6, 6 vs. 12, 12 vs. 18, 18 vs. 24 weeks); seven rats from each group were compared at each time point. CHP: cumene hydroperoxide.

Levels of lipid peroxidation in aorta increased at 12 weeks of a high-fat diet and remained high throughout the experimental period when compared to the CT group. In the HFD group, there was an increase in lipid peroxidation at 12 weeks when compared to 6 weeks (Figure 5).

### Systolic blood pressure

**Figure 6:**
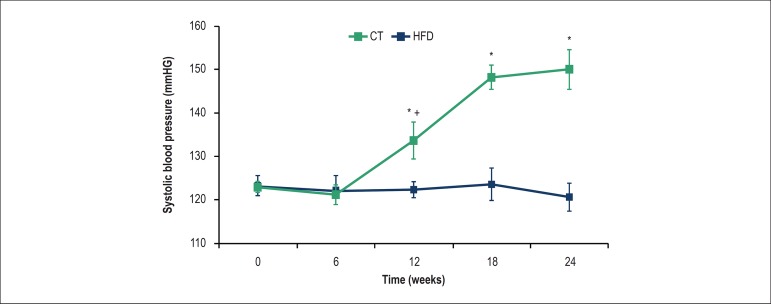
Systolic blood pressure in rats of control (CT) and high-fat diet (HFD) groups over 24 weeks. *P < 0.05, compared with CT group; + p < 0.05, within-group comparison (0 vs. 6, 6 vs. 12, 12 vs. 18, 18 vs. 24 weeks); seven rats from each group were compared at each time point

As shown in Figure 6, high-fat diet induced an increase in SBP at 12, 18 and 24 weeks in the HFD group when compared to the CT group. Moreover, a positive correlation was found between SBP and VAT (r = 0.756, p < 0.01) in the HFD, and no significant differences in blood pressure were found in the CT group.

### Alterations in the vascular structure

Table 2 shows that high-fat diet induced an increase in aortic medial thickness after 18 weeks and 24 weeks, and decreased the ID after 24 weeks in HFD compared to CT group (p < 0.05), resulting in an increase in the media thickness/lumen ratio after 18 and 24 weeks. In HFD group, there was an increase in the intima-media thickness after 18 weeks of high-fat diet, a decrease in the ID after 12 weeks, and an increase in the media thickness/lumen ratio in the aorta after 18 weeks of high-fat diet.

**Table 2:** Quantitative values obtained from morphometrical analysis of thoracic aorta thickness from control group (CT, n = 7) and high-fat group (HFD, n = 7) rats. Results are expressed as means ± SD. * P < 0.05, compared with CT group; + p < 0.05, within-group comparison (0 *vs*. 6, 6 *vs*. 12, 12 *vs*. 18, 18 *vs*. 24 weeks)

Weeks	Media Thickness (µm)		Internal diameter (ID) (µm)		Media:lumen ratio	
	CT	HFD	CT	HFD	CT	HFD
0	157.99 ± 7.18	157.88 ± 4.75	2830.64 ± 75.20	2832.64 ± 75.98	0.056 ± 0.00	0.056 ± 0.01
6	163.51 ± 7.51	163.64 ± 11.98	2967.21 ± 177.85+	2919.31 ± 145.46	0.054 ± 0.00	0.056 ± 0.00
12	162.82 ± 6.67	164.64 ± 9.64	2976.80 ± 167.73	2876.36 ± 99.89+	0.055 ± 0.01	0.057 ± 0.00
18	161.65 ± 9.95	178.20 ± 5.26 *+	2987.53 ± 156.18	2854.40 ± 133.40	0.054 ± 0.00	0.062 ± 0.00*+
24	164.21 ± 9.51	181.96 ± 9.73 *	3045.25 ± 168.01	2835.53 ± 167.74*	0.054 ± 0.00	0.064 ± 0.01*

## Discussion

To the best of our knowledge, this is the first study that has detected the time course of vascular function, vascular structure, oxidative stress and inflammatory status during the obesity progression in just one experimental model. Our findings showed that inflammatory state and endothelial dysfunction precedes the development of high blood pressure induced by high-fat diet. Obesity progression was associated with increased predisposition to pathological conditions and to common features of cardiovascular risk factors, including hypertension and endothelial dysfunction.^17^

The high-fat diet used in this study induced differences in adiposity between HFD and CT groups, validating our experimental model. The risk of developing obesity-related derangements is proportional to the degree of adiposity^18^ and, in particular, to the accumulation of fat in the visceral region.^19^ In this study, the HFD group had greater VAT mass at 6, 12, 18 and 24 weeks than the CT group.

In obesity, the inflammatory status is distinctive,^19^ and is characterized by low-grade inflammation, which results in tissue remodeling and systemic metabolic deterioration over time.^20^ Thus, detecting the time of increased inflammation is important for the development of therapeutic intervention.

Adipose tissue is fundamental to the development of inflammation by inducing the increase of pro-inflammatory cytokines, including TNF-α and IL-6,^21^ and a decrease in anti-inflammatory chemokines such as adiponectin.^22^ In addition, it has been described that TNF-α contributes to CRP elevation, which is a marker of low-grade inflammatory state, but also has a close relationship with dyslipidemia and endothelial dysfunction.^23^ In mice, the HFD induced an elevation of IL-6 after 2, 4 and 6 months,^24^ and an increase in plasma levels of pro-inflammatory mediators TNF-α, IL-6 after 15 weeks.^25^

In the present study, we detected that the levels of inflammatory cytokines TNF-α, IL-6 and CRP increased after 6 weeks in the HFD group and remained higher up to 24 weeks, while the adiponectin concentration was reduced and remained lower in the same period. These results indicate an early development of a low-grade inflammation state in this animal model. TNF-α is involved in the systemic inflammatory response and its levels are increased in the adipose tissue of obese mice compared with lean controls.^20^ On the other hand, adiponectin, which improves cardiovascular functions and has anti-inflammatory effects^22^ decreased after 6 weeks in HFD group and remained lower up to 24 weeks.

Obesity is also associated with an impairment of endothelial cell function and promotes endothelial dysfunction through an array of metabolic disorders including the accumulation of adipose tissue, high blood pressure, dyslipidemia and diabetes, which are linked to vascular oxidative stress.^25^ The endothelium comprises the inner lining of blood vessels, and forms the interface between the circulating blood and the vascular wall. It also acts as an endocrine and paracrine organ, which regulates vascular function by secreting a variety of trophic and vasoactive factors that regulate vascular tone, cell adhesion, smooth muscle cell proliferation and inflammation of the vascular wall.^8^

Endothelial dysfunction has a key role in the development of various cardiovascular diseases. In obesity, many factors could negatively affect the endothelium function, which include changes in blood pressure, glucose levels, lipid metabolism and inflammatory system, elevated levels of free fatty acids and oxidative stress, which in turn causes a reduction in the availability of NO.^26-28^

We observed that 6 weeks of high-fat diet was sufficient to induce endothelial dysfunction. Moreover, our results suggest that the impaired relaxation to acetylcholine observed in aortas from obese rats is related to a reduction of NO production. The HFD group showed the lowest serum concentration of NO at 6 weeks, which remained low up to 24 weeks. Consistent with our observations, various studies have shown obesity-induced impairment of endothelial function at different points of obesity development. Boustany-Kari et al.^29^ observed impaired endothelial function in rats fed for 11 weeks on a high-fat diet. In addition, 16 weeks of a high-fat diet in mice led to endothelial dysfunction and increases in systolic pressure in animals.^30^

Moreover, levels of TNF-α are strongly correlated with adiposity and diminished vasodilation in resistance arteries of rats, and IL-6 levels are proportional to adiposity whose elevations result in direct impairments of endothelial function.^31^ On the other hand, the decreased adiponectin levels are associated with dyslipidemia and cardiovascular diseases. Furthermore, adiponectin can upregulate NO production by modulation of Ser1177 phosphorylation through AMPK and, conversely, IL-6 and TNF-α decrease eNOS Ser1177 phosphorylation, resulting in diminished eNOS activity and less NO generation.^32^

In addition, we found a strong correlation between inflammatory cytokines (TNF-α, IL-6, CRP) and endothelial function (pD_2_).

Our findings are consistent with literature, showing that a high-fat diet treatment for 6 weeks was able to increase VAT. Interestingly, an inverse correlation was observed between VAT and endothelium function (pD_2_). In addition, the levels of these adipokines were altered at 6 weeks in the HFD group, confirming the concept of obesity-related endothelial dysfunction. We showed here that these events occur at an early stage of obesity development.

Obesity is also strongly associated with hypertension, which is a major risk factor for the development of coronary heart diseases. In fact, 79% of hypertension in men was a direct result of excess weight.^33^ Hypertension, characterized by chronic high blood pressure, has a multifactorial origin and an endothelial dysfunction can contribute to its genesis and maintenance.^5^ In the present study, the high-fat diet induced an increase in the SBP at 12 weeks and it continued to increase reaching the maximum values at 18 weeks. These results are in accordance with Boustany et al.^34^ that observed a rise in blood pressure, and increased activity of adipose and systemic renin angiotensin system after 11 weeks of high-fat diet in rats. The Framingham Heart Study reported a close connection between body fat levels and blood pressure in both men and women, and that adiposity emerged as a major factor which can be controlled and that contributes to hypertension.^34^ The same occurred in our study, which showed a strong correlation between SBP and VAT.

Interestingly, in the present study, structural alterations in the aorta occurred after a rise in blood pressure. It is well known that hypertension is associated with structural alterations in arteries that could contribute to maintaining hypertension.^35^ In addition, although not significantly, the media/lumen ratio starts to increase at 12 weeks, coinciding with the rise of blood pressure, and at 18 and 24 weeks, this increase becomes significant. Chen et al.^36^ found that the high-fat diet induced the increase in media thickness after 9 weeks. Our findings are in good agreement with these reports.

High-fat diet can also induce vascular pathogenesis, including effects on the aorta, leading to changes in vascular structure. Clinical and experimental studies have shown that increased body mass index is often associated with stiffening and increased arterial wall thickness.^37^ These alterations found in this study are important predictors of increased cardiovascular mortality.

Previous studies in animals suggested that hypertension is associated with an increased formation of reactive oxygen species (ROS) from all layers of the vascular wall.^38^ In agreement with these results, our findings showed an increase in lipid peroxidation (used as a marker of oxidative stress) in aortic rings at the same time that SBP increased, starting at 12 weeks. Moreover, Kobayasi et al.^30^ found a reduced antioxidant activity, increased local vascular inflammation and impaired endothelium-dependent relaxation in mice fed on a high fat diet at 16 weeks. The release of IL-6, mainly from abdominal adipocyte sources might have a pivotal role in the relationship between oxidative stress and endothelial dysfunction. IL-6 and TNF-α contribute to CRP elevation, a marker of low-grade inflammatory state, and also have a close relationship with endothelial dysfunction.^23^

As mentioned earlier, obesity is commonly associated with oxidative stress,^39^ which is able to modify vascular tonus by impeding NO bioavailability and/or signaling.^38^ We have observed that 6 weeks of high-fat diet decreased NO circulating levels without significant effects on aortic lipid peroxidation at this point of obesity progression. Thus, these results suggest that the decrease in circulating NO levels precedes the increase in oxidative stress. During the oxidative stress state, excessive production of ROS reduces the bioactivity of NO due to its rapid oxidative inactivation by the ROS superoxide (O_2_^−^).^38^

According to Victor et al.,^40^ while visceral fat stores expand, adipocytes generate increasing levels of ROS. In the present study, the high-fat diet induced the accumulation of abdominal fat that could trigger lipid peroxidation in the aorta at 12 weeks, which persists up to 24 weeks.

One limitation of this study was the fact that visceral fat mass was evaluated by dissection of adipose tissue. Dual-energy X-ray absorptiometry (DXA), the gold standard method for assessment of body fat mass, would provide more comprehensive data of body composition; but, unfortunately, the method could not be performed in this study.

Our data suggest that even at early stages of development, obesity (6 weeks) can trigger chronic inflammation and impairment of endothelial function. This impairment appears most closely related to inflammatory cytokines and expansion of VAT.

## Conclusion

In conclusion, development of obesity first led to a reduction of endothelial function, which continued to decline over the weeks, and to systemic inflammation, followed by an increase in blood pressure, lipid peroxidation and changes in aortic structure. Our work is relevant in showing the relationship of obesity with chronic inflammation, endothelial dysfunction and hypertension. Despite many studies in this area, the results we found are a further step towards to the development of therapeutic strategies to prevent these abnormalities.
