# Erratum

**DOI:** 10.5935/abc.20190004

**Published:** 2019-01

August issue of 2018, vol. 111(2), pages 230-289

Consider correct the following page numbering of contents for the items: for page 4, consider 231; respectively: 5, 234; 6, 235; 7, 236; 10, 239; 11, 240; 13, 242; 15, 244; 16, 245; 18, 247; 19, 248; 20, 249; 21, 250; 22, 251; 23, 252; 29, 258; 30, 259; 33, 262; 34, 263; 35, 264; 37, 266; 39, 268; 41, 272; 44, 273; 46, 275; 47, 276; 48, 277; 49, 278; 50, 50; 51, 280. For the items indicated with the pages 24-28 and 31-32, consider: 4.1.2, 4.1.2.1, 4.1.2.2, A) Ciclosporina - page 253; B) Tacrolimus, 4.1.2.3, A) Azatioprina, B) Micofenolato, 4.1.2.4, A) Sirolimus - page 255; B) Evrrolimus, 4.1.3, 4.2, 4.2.1, 4.2.2, 4.2.2.1 - page 256; 4.2.2.2 - page 257; 4.2.3 - page 258; 4.2.3.1 - page 258; 4.2.3.2 - page 258; 4.3.2, 4.3.3, 4.3.3.1 - page 260; 4.3.3.2 - page 261. On page 251, the correct numbering of the items “Gravidade e Biomarcadores”, respectively, is 3.4.4 and 3.4.5. On page 252, the correct numbering of the “Estratégias de Prevenção e Tratamento” item is 3.4.6.

August issue of 2018, vol. 111(2), pages 290-341

Consider correct the following page numbering of contents for the items: for page 3, consider 292; respectively: 4, 293; 5, 294; 6, 295; 7, 296; 8, 297; 10, 299; 12, 301; 13, 302; 14, 303; 15, 304; 16, 305; 17, 306; 18, 307; 19, 308; 20, 309; 21, 310; 22, 311; 23, 312; 24, 313; 25, 314; 28, 317; 29, 318; 30, 319; 31, 320; 32, 321; 33, 322; 24, 323. The item “Referências” was included. In the content, page 325.

September issue of 2018, vol. 111(3), pages 436-539

In the content, items “5.3.4.4. Reposição volêmica” and “5.3.4.5. Diurético” were removed, because they are repeated. The item “5.3.4.6. Betabloqueadores” has been corrected to “5.3.4.4.”. The item “5.3.4.5. Vasoconstritores e inotrópicos” was included.

Consider correct the following page numbering of contents for the items: for page 4, consider 441; respectively: 5, 442; 6, 443; 7, 444; 8, 445; 9, 446; 10, 447; 11, 448; 12, 449; 13, 450; 14, 451; 15, 452; 16, 453; 17, 454; 18, 455; 19, 456; 20, 457; 21, 458; 22, 459; 23, 460; 24, 461; 25, 462; 26, 463; 27, 464; 28, 465; 29, 466; 30, 467; 31, 468; 32, 469; 33, 470; 34, 471; 35, 472; 36, 473; 37, 474; 38, 475; 40, 477; 41, 478; 44, 481; 45, 482; 47, 484; 51, 488; 52, 489; 53, 490; 54, 491; 56, 493; 57, 494; 58, 495; 59, 496; 60, 497; 61, 498; 62, 499; 63, 500; 64, 501; 67, 504; 70, 507; 71, 508; 73, 510; 74, 511, 75, 512; 76, 513; 77, 514.

On page 514, the correct numbering of the items “Reposição volêmica”, “Diuréticos”, “Betabloqueadores” and “Vasoconstritores inotrópicos”, respectively, is 5.3.4.2, 5.3.4.3, 5.3.4.4 and 5.3.4.5.

June issue of 2018, vol. 110(6), pages. 558-567

In the Original Article “Endothelial Dysfunction and Inflammation Precedes Elevations in Blood Pressure Induced by a High-Fat Diet”, pages 558-567, by authors Jorge Camargo Oishi, Cynthia Aparecida Castro, Karina Ana Silva, Victor Fabricio, Evelin Capelari Cárnio, Shane A. Phillips, Ana Claudia Garcia de Oliveira Duarte, Gerson Jhonatan Rodrigues, the correct affiliation of Dr. Shane A. Phillips is University of Illinois at Chicago, Illinois - USA.

